# Case of Gastrotomy—Read before the Tennessee State Medical Society, by Dr. I. E. Manlove, and Forwarded, by Vote of the Society, for Publication in the Boston Medical and Surgical Journal

**Published:** 1845-09

**Authors:** 


*Case of Gastrotomy.*—Read before the Tennessee State Medical Society by Dr. I. E. Manlove, and forwarded, by vote of the Society, for publication in the Boston Medical and Surgical Journal.

On the 7th of July, 1844, I was called to see Alfred, a colored boy, aged 17 years. He complained of some general uneasiness of the abdomen, was laboring under febrile excitement, pulse 110. Learned that he did not recollect having a passage from the bowels in 12 or 15 days. On the 4th had walked several miles to a barbecue, and probably had indulged freely in eating. He had taken Epsom salts and castor oil; also several enemata had been administered by his master. I bled him "*ad deliquium animi*," gave him a general warm bath, and directed 4 grains of calomel and ½ grain of opium every four hours, until three portions should be taken, to be followed by castor oil and spts. turpentine.

8th.—Medicine had been all taken; no evacuation of the bowels; had vomited once, throwing up the medicine. Pulse

120. Bled him, administered a stimulating enema, and directed calomel and opium as on the previous day. Visited him again in the afternoon. Condition found to be the same; no evacuation. Spent the night with him, and made every effort I could to procure evacuation of the bowels, but they all proved ineffectual. Vomited several times during the night. Pulse 120 and feeble. Abdomen tympanitic.

9th.—Dr. Ford was called in consultation. His condition remained the same, except that all the symptoms were now growing more and more alarming, with the certainty that death must speedily ensue without relief. Flexible tubes were introduced as far as possible into the intestines, and stimulating articles were thrown up so as literally to fill the lower bowels. These were all soon thrown off without any appearance of fæces. About 60 grains of tart. antimony were dissolved in water and introduced at two injections, with little or no influence on the general system. An emetic also of ipecac. was administered; emesis was readily produced, but no alteration in the symptoms. Being now night, it was thought advisable to wait on the means which had been used until morning.

10th.—Abdomen enormously distended; difficulty of breathing; extremities cold; pulse very feble and quick; countenance anxious, no evacuation. Gastrotomy was considered the only possible means of even prolonging his life; and although the operation promised but little benefit, yet the certainty of death without it, justified us, in our estimation at least, in undertaking its performance. An incision was made in the median line, commencing about two inches below the umbilicus, and extending down towards the pubis four or five inches. The peritoneum and bowel along the lower half of the incision had formed a most intimate adhesion, and in cutting through the former an opening of about one fourth of an inch in extent was made into the latter. From the opening there proceeded large quantities of flatus and liquid fæces, as well as the oil and turpentine which had been taken. On further examination, it was discovered that the intestines were united to the peritoneum by extensive adhesions at various points within reach of the finger and probe. The wound was closed by sutures and adhesive straps, except the opening into the intestine. The amendment in all the symptoms in one hour was astonishing; the extremities became warm, the pulse slower and fuller, and during the morning he was able to fan himself, the weather being excessively warm. On the next day his appetite was good, and he continued to improve and to discharge the contents of the bowels through the artificial anus until the 17th day after the operation, when the bowels acted naturally, the opening having nearly closed.

It will be proper to state, that about six months before his present illness, the boy received an injury from the falling of a piece of timber on the abdomen. The hurt caused him to keep his bed several weeks, and hence, no doubt, the adhesions which were discovered in the operation. The boy is now well (nine months after the operation), and is present for the inspection of the members of the Society.

That there was mechanical obstruction of a serious nature in this case, cannot be doubted; but it is a matter of much difficulty to determine its character. The opinion to which we incline is, that it was dependent on the retarded peristaltic motion of the intestines, which was the effect of the adhesions formed between the peritoneum and the bowels. Now we think it probable that from inattention to the regular evacuation of the intestinal canal, to imprudence in diet, or some other similar cause, accumulations of fæces may have taken place about the point of greatest adhesion, and the already retarded peristaltic action forming the still greater accumulation of matter, until the bowel was forced to bend on itself, forming a barrier which no ordinary means could overcome. This supposition is favored by the long-continued constipation under which the patient had labored, as well as by the result of the operation. It will be observed that passages per "vias naturales," did not take place for seventeen days, in which time it is probable that the accumulation was removed through the artificial opening, by being gradually dissolved and discharged, and so soon as that was effected to a sufficient extent, the bowels acted naturally. It is, however, of not so much importance to know the exact condition in this case, as to deduce inferences from it which may, perhaps, bear on cases hereafter to be treated.

The medical books abound with the detail of cases of obstructed bowels, and no doubt many of us have met with such in practice, in which all the remedies have been used in regular gradation, from the mildest laxative to the most drastic purgative, with all the other cathartic means, and the patients have died without an evacuation from the bowels. And the concurrent testimony of the faculty has tended to the conviction that in this condition nothing more should be done than has been done—that a respite from remedies should be allowed for the "settlement of worldly concerns and for the preparation of the spirit against the inevitable hour."

We would consider such a case not only justifying, but loudly calling for the interference of surgery. An operation which would discharge the contents of the bowels above the obstructed point, through an artificial anus, would tend to postpone the fatal hour, and perchance place the unhappy patient in that condition in which the powers of the system might restore him to health. Back to the earliest date of surgery may be traced the fear of peritoneal inflammation, as good and sufficient reason against the performance of the operation of gastrotomy. And we would hazard the suggestion that that fear has been proved by facts, belonging to this age, to be, if not groundless, at least stripped of much of its terror. We would not be understood to say that an artificial anus should be made in every case of gastrotomy—but only in those where the obstruction cannot be relieved by a change in the position of the intestinal tube. The necessity for opening the intestine should be judged of after the incision has been made in the abdomen and the cause of obstruction examined.

In reading, lately, the detail of the case of Hon. H. S. Legare, we were forcibly struck with its almost exact description in many points with Alfred's case, at the head of this paper. In cases of torsion, as well as of intussusception of the bowels, we would with deference suggest the propriety of the operation of gastrotomy, to relieve the cause of mechanical obstruction, as affording the only means of saving the patient's life—and respectfully as we would consider the fear of peritoneal inflammation, we do not deem it of so much importance as the inevitable death of the patient without the operation. In the operation for strangulated hernia the peritoneum is wounded; and if it be performed skilfully and soon enough, the danger of peritoneal inflammation is not considered a prominent feature in the case. Desault says that a patient should never be lost on whom the operation is performed before the taxis is used. The chief difficulty in such cases would be to determine the point of time when ordinary remedies will be of no further avail, and still organic lesion has not advanced too far to preclude the possibility of relief by operation. And we say, with Hey, that we might often regret that the operation is performed too late, but seldom that it is performed too early.

In support of these views as far as one instance will go, we will here mention a case which may possibly be brought before the Society in another form. Our respected friend, Dr. Wilson, of this county, was called to attend, in conjunction with several others, a negro man who was supposed to be laboring under intussusception. All remedies had been used to procure evacuation of the bowels which ingenuity could suggest, but in vain; and the patient was reduced to the last point of life. Gastrotomy was determined to be the only means of affording relief. It was performed by Dr. W., and the intestines drawn out of the cavity of the abdomen until the point of obstruction was arrived at. About one inch of the ileum was found to be invaginated; and the attempt to relieve the intussuscepted portion, discovered the fact that adhesions had been formed between the receiving and received parts of the intestine. The adhesions were dissected loose, and the bowels returned into the abdomen. Natural passages immediately took place, and the patient was rapidly restored to perfect health. It is not unreasonable to suppose the chances of recovery would have been much enhanced, had the operation been performed before the adhesions were established.

If the chief dread in gastrotomy, in such cases, be peritonitis, it may be removed somewhat by the reflection that that disease is combated successfully every day by the well-established means which we all have at hand. And if it supervene after an operation, may not the same means be successful? And when we consider the importance of the operation, as affording the only chance of recovery to the patient, we may surely conclude that it is the smaller of two evils, and justifiable in its adoption. We have but little doubt that experience will show that this operation may be performed with as little danger to the patient, and with as great prospect of relief, as that of lithotomy, and many others equally important. It is apparent however, that the success of the operation depends much on the time of its performance. If delayed until disorganization of the intestinal tube is established, failure will await us almost inevitably. If performed sufficiently early, we would expect to be successful oftener than otherwise.—*Ibid.*

